# Challenges in Using Recommended Quality of Life Measures to Assess Fluctuating Health: A Think-Aloud Study to Understand How Recall and Timing of Assessment Influence Patient Responses

**DOI:** 10.1007/s40271-021-00555-7

**Published:** 2021-12-02

**Authors:** Sabina Sanghera, Axel Walther, Tim J. Peters, Joanna Coast

**Affiliations:** 1 Health Economics Bristol (HEB), Population Health Sciences, Bristol Medical School, University of Bristol, 1-5 Whiteladies Road, Bristol, BS8 1NU UK; 2 Bristol Cancer Institute, University Hospitals Bristol NHS Foundation Trust, Bristol, UK; 3 Population Health Sciences, Bristol Medical School, University of Bristol, Bristol, UK

## Abstract

**Background:**

It can be challenging to measure quality of life to calculate quality-adjusted life-years in recurrent fluctuating health states, as quality of life can constantly change. It is not clear how patients who experience fluctuations complete measures and how assessment timing and recall influence responses.

**Objective:**

We aimed to understand how patients with fluctuating health complete widely recommended and commonly used measures (EQ-5D-5L, EORTC QLQ-C30 and SF-12) and the extent to which the recall period (‘health today’, ‘past week’ and ‘past 4 weeks’) and timing of assessment influence the way that patients complete these questionnaires.

**Methods:**

Twenty-four adult patients undergoing chemotherapy for urological, gynaecological or bowel cancers in the UK participated in think-aloud interviews, while completing the measures, completed a pictorial task illustrating how quality of life changed during the chemotherapy cycle and took part in semi-structured interviews. Transcripts were analysed using constant comparison.

**Results:**

Patients were consistent in describing their quality of life as changing considerably throughout a chemotherapy cycle. The shorter recall period of ‘health today’ does not adequately represent patients’ quality of life because of fluctuations, patients remarked they could give a different answer depending on the timing of assessment, and many struggled to combine the “ups and downs” to answer measures with longer recall (‘past week’ and ‘past 4 weeks’). Across all measures, patients attempted to provide averages, adopt the peak-end rule or focus on the best part of their experience. Patients commonly used more than one approach when completing a given questionnaire as well as across questionnaires.

**Conclusions:**

Patients who experience recurrent fluctuations in health are unable to provide meaningful responses about their quality of life when completing quality-of-life measures due to the recall period and timing of assessment. The use of such responses to calculate health state values in economic evaluations to inform resource allocation decisions in fluctuating conditions must be questioned.

**Supplementary Information:**

The online version contains supplementary material available at 10.1007/s40271-021-00555-7.

## Key Points for Decision Makers

**Table:** 

Patients who experience fluctuations in health do not feel their experience of treatment is well reflected when researchers use recommended quality of life questionnaires.
Patient responses could be markedly different depending on the timing of the assessment.
Measures with longer recall periods result in patients attempting to construct averages and/or focus on the best or worst part of their experience.
Patients do not complete any measure systematically, as they adopt multiple approaches for the same recall. The value of current approaches of eliciting patient experience and the use of these responses to provide recommendations on cost-effectiveness must be questioned.

## Introduction

In many chronic conditions, health states are relatively constant. In these conditions, patients may experience fluctuations, but they do not lead to large recurrent changes in quality of life. There are, however, many examples of conditions that have fluctuating health states where patients repeatedly experience a sudden change in quality of life or where changes in quality of life are cyclical. These are referred to here as recurrent fluctuating health states. These changes can be either predictable or unpredictable. One such example, used as an exemplar in this paper, of fluctuating health states results from side effects from chemotherapy, commonly employed for the treatment of cancer. Worldwide, 17 million new cases of cancer were diagnosed in 2018, and in the UK, approximately 28% of people diagnosed with cancer are treated with chemotherapy [1, 2]. A course of chemotherapy is made up of several cycles, where a cycle is the period between one treatment and the next. A cycle typically lasts 3–4 weeks, with treatment on the first day(s) and a subsequent rest period; commonly four to eight cycles of chemotherapy are used [3], although treatment can be ongoing. The side effects from treatment are a significant health burden [4] and tend to occur early in each cycle followed by recovery during the rest period [5]. Over a course of chemotherapy with several cycles, patients are likely to experience marked cyclical fluctuations in quality of life. Such fluctuations would be expected to a greater or lesser degree with all chemotherapy.

Many international institutes recommend that quality-adjusted life-years (QALYs) are used as the outcome to report the impact of health interventions on length and quality of life [6–11]. To capture quality of life, several measures exist. The most frequently recommended measures include EQ-5D [12], SF-6D (by converting scores on the SF-12 or SF-36 [13, 14]) and the Health Utilities Index [6, 7, 10, 11, 15–18]. These measures have been validated for use in many conditions, but it is unclear if the measures themselves and the way they are used is valid for conditions or treatments that cause recurrent fluctuating health states. Possible reasons why the measures may not be valid in the context of recurrent fluctuating health states are: (1) the standard recall periods of the questionnaires (‘health today’ for EQ-5D and ‘past 4 weeks’ for SF-12 and SF-36) and (2) the timepoint in a patient’s fluctuating health state at which a measure is administered. There is a lack of understanding about how patients complete these measures when health is fluctuating and how the timing of assessment and the recall period influence completion. These points, coupled with the usual assumption when calculating the QALY that the change in health over time is constant between measurement timepoints (i.e. linear interpolation of scores), could lead to systematic bias and misleading economic recommendations in conditions with recurrent fluctuating health states; the precise pathways from which such bias may result are detailed in earlier work that explores issues around recall and timing [19]. For example, in the context of cancer chemotherapy, measuring health always at day 1 of the cycle with a recall period of today may mean systematically biasing measurement to a ‘good day’ in the cycle but using a 4-week recall period may leave patients systematically including the first week of a 3-week cycle twice in every calculation—if they are even able to make this judgement.

To understand how patients with fluctuating health states complete measures and then how the recall and timing of assessment influence responses, think-aloud interviews were conducted while patients completed three measures that are widely recommended and commonly used: EQ-5D-5L, SF-12 and the cancer-specific measure EORTC-QLQ-C30. Think-aloud interviews can generate understanding around how respondents interpret time horizons and the choices they make when providing a response. These interviews have successfully been used in this context; the verbal information provided whilst completing the task yielding insights into questionnaire completion [20–24]. This paper forms part of a broader study, which has the overarching aim to assess the impact of cyclical fluctuating health states on economic evaluation results [19].

## Methods

Think-aloud interviews were conducted using three questionnaires followed by pictorial tasks and semi-structured interviews with patients undergoing chemotherapy for cancer. Three questionnaires with varying time periods commonly used in economic evaluations of chemotherapy treatments were chosen: EQ-5D-5L with five dimensions (mobility, self-care, usual activities, pain and anxiety/depression) and a recall period of ‘health today’ for all questions [12]. SF-12 has seven domains: general health, physical functioning, role limitation, social functioning, pain, mental health and vitality [25]. The recall period for nine questions in the SF-12 questionnaire refer to the ‘past 4 weeks’, the recall period for three remaining questions refer to ‘health now’ and ‘in general’. Only seven of the 12 questions of SF-12 are mapped onto SF-6D to obtain QALYs; only one of these questions does not refer to the ‘past 4 weeks’ [13]. An alternative recall of ‘past week’ is available, but this is less commonly used in practice and thus the commonly used ‘past 4 week’ recall was used in this research. EORTC-QLQ-C30 is a cancer-specific questionnaire primarily focused on symptoms. The measure has a recall period of the ‘past week’, but this also varies as the first 5 out of 30 questions do not refer to a specific recall period [26].

### Sampling

Patients undergoing chemotherapy for cancer were recruited during a visit to one oncology department. Patients were initially approached face to face by the researcher, an oncologist or a research nurse. Patients were eligible if they were receiving treatment for any urological, colorectal or gynaecological cancer with any cyclical chemotherapy regime, and able to communicate in English. Children or vulnerable adults were excluded, and patients were not approached during their first cycle of chemotherapy.

Patients were consecutively recruited until little new information on recall was observed in the interviews. An approximate a priori sample size of 25 patients was thought likely to be adequate to identify important themes from the interviews and to draw conclusions about the completion of the three quality-of-life measures for patients undergoing chemotherapy. Previously published think-aloud studies have used sample sizes with a range of 10–34 participants [27, 28].

### Data Collection: Interview Conduct

Face-to-face think-aloud interviews were conducted in English (by SS, a female academic health economist with a PhD who received grant funding to conduct the research, and had prior experience and training in conducting interviews) at the patient’s convenience (either in a university building or the patient’s home). Field notes were taken. The patients were aware of the purpose of the research and had only met the interviewer prior to the interview as part of the recruitment and consent process. Each patient took part in one interview, which began with a warm-up exercise. Patients were asked to think aloud as they completed three questionnaires. As patients were providing responses to items on a questionnaire their answers were prompted. The think aloud was followed by a pictorial task where patients drew on a graph (with a 0–1 quality-of-life scale on the y-axis and time during one chemotherapy cycle on the x-axis) how their quality of life changed during one cycle of chemotherapy, and finally a semi-structured interview to clarify any uncertain answers. Interviews for different individuals were held at different points in the cycle.

### Data Analysis

Interviews were recorded on a digital recorder and transcribed verbatim. The transcripts were not returned to patients for comments or corrections. Interviews were analysed using constant comparative approaches [29] with early transcripts being independently open coded by two reviewers (SS and JC). Coding involved detailed reading and re-reading of each transcript and labelling each segment of text to summarise the point. All the codes were then reviewed to identify links between them and create broader categories or ‘themes’ and subcategories. Common themes were identified and shared with the broader research team, and a hierarchical coding framework was generated incorporating this open coding as well as deductive themes arising from the data. Remaining transcripts were coded (by SS) using NVivo and the agreed framework. Analytic accounts [30] were then used initially to compare new data to existing data, and then to categories generated through the coding process. An analytic account consisted of a detailed description of the data and relevant quotes for each of the categories using three transcripts at a time (see Electronic Supplementary Material [ESM 1], for an extract from an analytic account). The process of writing analytic accounts forces the analyst to be explicit in their comparisons, describe data in context and make connections between categories and sub-categories. The analytic accounts from all transcripts were then combined and refined to provide more explanatory accounts of the data based on the focus of the analysis, which was on adherence to recall periods, construction of averages over fluctuating experiences, recalling the worst or best point of the chemotherapy cycle and the influence of timing of assessment. Quotes are presented verbatim with the use of ellipses to represent missing text; phrases or words that do not add meaning are excluded without the use of ellipses.

## Results

**Fig. 1:**
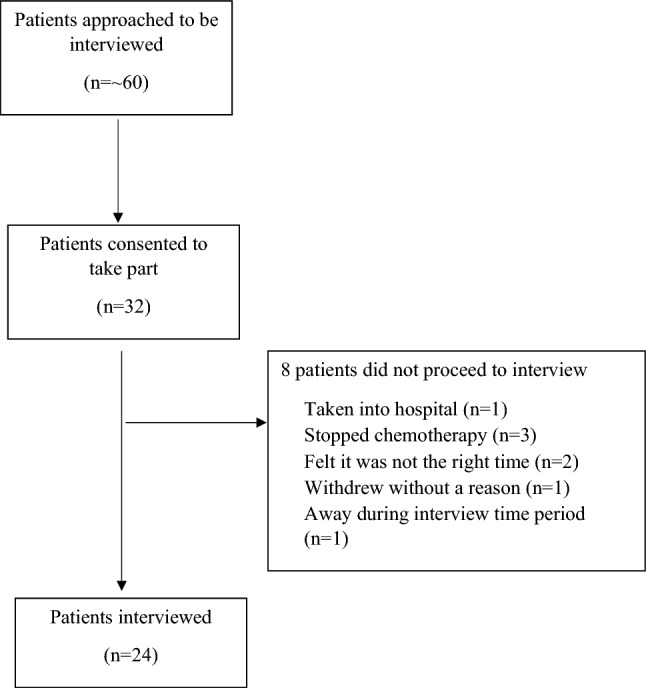
Flowchart of included patients

Between July 2018 and September 2018, approximately 60 patients were approached, and 32 agreed to take part. Twenty-four patients proceeded to interview (see Fig. 1), three patients had a partner present at the time of the interview. At this point, saturation within the predicted and emerging themes was achieved as no new information or themes were observed in the data and recruitment was stopped (see Table 1 for patient characteristics).

**Table 1:** Characteristics of included patients

Characteristic	*N* (%)
Age, years	
< 50	1 (4)
50–69	12 (50)
> 70	11 (46)
Sex	
Female	11 (46)
Male	13 (54)
Cancer type	
Gynaecology	9 (38)
Urology	7 (29)
Colorectal	8 (33)
Treatment intention	
Curative	8 (33)
Palliative	16 (67)
Frequency of chemotherapy	
3 weekly	21 (88)
4 weekly	3 (12)
Chemotherapy week at time of interview	
1	9 (38)
2	7 (29)
3	8 (33)
4	0 (0)

Twelve interviews took place in the patient’s home, 11 in a university building and one at a hospital. The average duration of the interviews was 44 min (range 21–112 minutes) and all patients answered all three questionnaires, two patients struggled with the pictorial task as they did not feel comfortable or able to draw how their quality of life changed and preferred to verbalise the change in quality of life instead. The relationships between the themes that were the focus of the analysis, as outlined previously, were reorganised and merged into four overarching key themes: patterns of quality of life, recall, influence of timing of assessment and how patients respond to questions when health is fluctuating.

### Patterns of Fluctuating Quality of Life

**Fig. 2:**
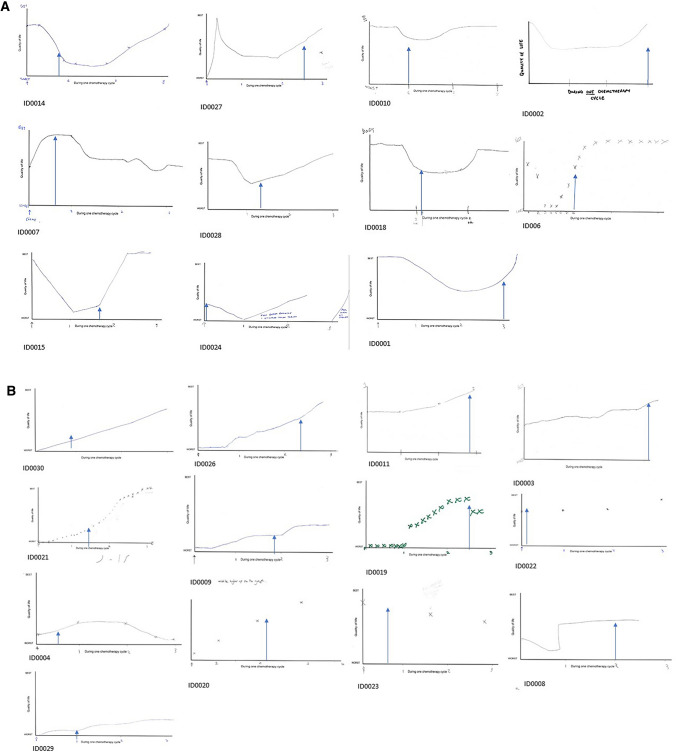
**a** Cyclical fluctuations in quality of life. Patient completed figures to illustrate how their quality of life changed over time (*x-axis*) during one cycle of chemotherapy. **b** Low/moderate quality of life that improves overtime. Patient completed figures to illustrate how their quality of life changed during one cycle of chemotherapy. The *arrow* indicates the point at which the interview took place

All patients were consistent in describing their quality of life as changing throughout the chemotherapy cycle. For many, the first few days after chemotherapy were described as having a relatively high quality of life, but this was followed by lows for the remainder of the first week and second week of the cycle, where they might struggle to do anything in a day. In the final week, patients described improvements to levels that made them feel back to ‘normal’ as they then approached their next cycle of chemotherapy (Fig. 2a, pictorial task).

***ID0018****: I have the chemo and it’s okay for a few days, then I’m not too good, and then I gradually improve (…) by next week [the final week before treatment], I will feel a lot better … [female, >70 years old, colorectal cancer, second week of chemotherapy cycle]*

***ID0001****: The first few days after having had the treatment, I don’t notice any lack of energy. (…) slowly through the first week, I can see my energy levels actually are decreasing, and then the second week, they are pretty constant at that lower level. Then they pick up again towards the end of that week and then all throughout the last week. (…) so by the time I come back for the chemo, I feel absolutely fine. [female, 50–69 years old, gynaecological cancer, third week of chemotherapy cycle]*

Whilst still describing their quality of life as changing throughout the cycle, some patients reported having a relatively low or moderate quality of life at the start of the treatment which, in most cases improved substantially overtime. The extent of the changes in quality of life and the level of quality of life at the start of treatment did not appear to depend on cancer type, treatment type, sex, age, cycle number or whether they were receiving adjuvant or palliative treatment (Fig. 2b, pictorial task).

***ID0019:*** *The worst time for me is the first week … [female, 50–69 years old, gynaecological cancer, third week of chemotherapy cycle*]

***ID0009****: I think it generally settled down to the fact that it did do what [the consultant] had always said, or what everyone always told you, that you feel ill the first week, slightly better the second week – not fine, but better – reasonable by the third week. [female, 50–69 years old, gynaecological cancer, first week of chemotherapy cycle]*

### Recall

#### Focusing on Recall

There was a tendency for patients to skim read the questionnaire instructions where some noticed the recall period and others appeared to miss it. However, because of the fluctuations in symptoms, it was very common to see patients start to complete a question and then refer back to the instructions to identify over what period they needed to answer the questions. The amount of introductory text heavily influenced whether the recall period was noticed, as lengthy text with the recall period provided in amongst the text resulted in fewer people noticing the recall.

#### ‘Today’, ‘Past Week’ and ‘Past 4 Weeks’

The influence of the fluctuating health symptoms was explored across three different recall periods: ‘today’ (EQ-5D-5L), ‘past week’ (EORTC-QLQ-C30) and ‘past 4 weeks’ (SF-12). When completing questions with a short recall period, such as ‘today’, it was clear that because of the constant changes in quality of life, a response over such a short timeframe did not reflect quality of life over the “whole cycle” of chemotherapy. Patients, themselves, questioned the appropriateness of this framing.

***ID0010****: That has got to be for today, has it? Particularly for today? ... I do normally have a few problems in walking about. [male, 50–69 years old, urological cancer, third week of chemotherapy cycle]*

***ID0006****: Well, I mean it’s easier to respond for today, but that might not be what you want. You might want things about the whole cycle, I don’t know, it depends what you want. [female, >70 years old, gynaecological cancer, second week of chemotherapy cycle]*

The recall period of ‘past week’ raised similar problems to ‘health today’, as patients commonly explained that this time period was not “representative of the whole chemotherapy cycle”.

***ID0014****: … it’s easy to lose track of that particular timescale, because you’re tending to think of yourself as, I’ve been on chemotherapy now for four months, and there are ups and downs in the chemotherapy, and so you might tend to answer a little bit as ****how have I been overall, and actually answering in terms of just the last week is not necessarily representative of the whole chemotherapy cycle****. When I get to the end of it, my last week before the next chemotherapy, I’m generally sort of, not okay, but I’m a lot better. So there is quite a variation between weeks one, two, and three, in my particular chemotherapy cycle. [male, 50–69 years old, urological cancer, first week of chemotherapy cycle]*

The longest recall period of ‘past four weeks’ also caused difficulty because of fluctuating symptoms. The problems were not due to the recall failing to represent the whole chemotherapy cycle, instead, patients struggled with remembering changing symptoms over a 4-week period and then trying to amalgamate the “ups and downs” to provide an answer. Consequently, questions were not answered systematically.

***ID0008****: Thinking about four weeks, I can just about remember what I've done the last week. (Laughter) So four weeks is quite a long time to think through how things have been. Particularly if there have been ups and downs. [female, 50–69 years old, gynaecological cancer, second week of chemotherapy cycle]*

Some patients even changed the 4-week recall period because it was difficult to answer the questions due to the mismatch in the 3-weekly cycle of the chemotherapy and the 4-week recall period of the questionnaire.

***ID0010****: That is difficult. I’m not really sure about that to be honest. Over the past four weeks, with that it sort of goes up and down …. Before that, it would have been about a week before I went to the hospital that I would have felt a bit low. It [the cycle] is every three weeks, so you can’t really say four weeks. [female, >70 years old, gynaecological cancer, first week of chemotherapy cycle]*

***ID0006****: The past four weeks? … If you call it three weeks, and I’m saying that the second weeks not too bad, the third weeks almost normal, that’s why I’m calling it some of the time. [female, >70 years old, gynaecological cancer, second week of chemotherapy cycle]*

### Influence of Timing of the Assessment

In addition to the recall period, the responses were clearly influenced by the timing of the assessment because of changing symptoms. The patients talked about giving a different answer to the question depending on the point at which they were assessed in their cycle.

***ID0001****: You ask me today, you’ll get one answer, and ask me tomorrow, you might get a different answer altogether, even if it’s in the same week. [female, 50–69 years old, gynaecological cancer, third week of chemotherapy cycle]*

***ID0014****: [immediately after treatment] that’s the period when I would really not do very much at all. Then when I get to the end of the first week … or the end of 10 days usually, I’m beginning to feel a bit better… and then, the last week of the cycle I’m … beginning to feel back to normal … ****So for me, I could give you a different answer in each part of that cycle**** … [male, 50–69 years old, urological cancer, first week of chemotherapy cycle]*

***ID0020****: today how much I can do compared to how much I could do say five days after chemo, I would say today I’ve only got slight problems compared to a different time in the cycle [female, <50 years old, gynaecological cancer, third week of chemotherapy cycle]*

The timing of assessment was clearly shown to impact on the quality of life responses provided when questions with shorter recall periods such as ‘today’ and ‘past week’ were used. Because of the longer time period of the ‘past 4 weeks’ recall period, patients were less likely to mention the timing of the assessment, but it did seem to influence what they focused on when answering questions (see Sect. 4).

The combination of a short recall period ‘today’ and timing of assessment could cause quality of life to be overestimated if patients happened to have a “good day” at the time of assessment or it could be underestimated if they were having a “bad day” at the time of assessment.

***ID0007****: This isn’t a typical day, no. A typical day would be I get on with things a lot slower than I would normally, but I look forward to enjoying the day, going out, doing things, being positive. Today is definitely different because I believe it’s … affected me, what it has done. You lose all your nutrients, don’t you, and your salts and that in your body? So, I’m very shaky today. So, I would say that’s why I feel differently than I would on a normal day. [female, 50–69 years old, gynaecological cancer, second week of chemotherapy cycle]*

Similarly, when answering questions about the ‘past week’, patients’ responses appeared to be dependent on timing of assessment. If the interview took place at the beginning or end of the first, second or third week post-chemotherapy or they did not experience a change in quality of life, patients could more readily complete the questionnaire, despite the answers not reflecting their quality of life over the whole cycle.

***ID0003****: When I think back over the last week, there hasn't been really any change. [male, >70 years old, colorectal cancer, third week of chemotherapy cycle]*

***ID0026****: The last week [recall] is the easiest because it's been alright. It's been the same. [male, >70 years old, urological cancer, third week of chemotherapy cycle]*

***ID0018****: if I did the questionnaire this week, I would have to say that I was not hungry and, you know, all these other things I’ve said. Hopefully, if it was next week, I could say I’ve improved a lot, I’m eating more and I’ve got a little bit more energy and my social life has improved. So, yes, I think separate things for separate weeks is quite a good idea. [female, >70 years old, colorectal cancer, second week of chemotherapy cycle]*

However, when completing the same questions, if a change was experienced or the questionnaires were completed in the middle of the first, second or third week post-chemotherapy, the changing symptoms seemed to lead to difficulties in completion because of the timing of the assessment and changing symptoms over that time period:

***ID0021****: During the past week. It’s kind of the middle, the last seven days will kick into just when I was feeling rough last week because I’ve only felt good the last two days. Put that into a frame of reference about where I am in the cycle, where seven days was. I’ve only been feeling good for two or three days. I have my chemo on a Monday, so I mark the weeks off on a Monday. Obviously, it changes gradually throughout the term [cycle]. I can mark a Monday and it’s always the first week is really bad. The second week is okay and the third week is even better. That’s not obviously the case, because it’s not like that. A definite marking of this second week was this. If this questionnaire asked at the end of the first week or the end of the second week, it would clear this is when it’s going to work. [male, <50 years old, colorectal cancer, second week of chemotherapy cycle]*

### How do Patients with Fluctuating Symptoms Respond to Questions?

#### Correct Timeframe

Patients most commonly provided answers that appeared to be most closely aligned to the recall period when answering questions about their health ‘today’. This also occurred in many cases when patients responded to questions about the ‘past week’, but less commonly. This may be because of the shorter instructions or the ease of referring to ‘today’ when symptoms are less likely to change to the same extent as over the past week or past 4 weeks.

***ID0008****: ‘Usual activities’. This is just today, isn’t it? Yes. No problems today. [female, 50–69 years old, gynaecological cancer, second week of chemotherapy cycle]*

***ID0006****: Today, so that’s today? Okay, your health today. I would say I have slight problems in walking about today. [female, >70 years old, gynaecological cancer, second week of chemotherapy cycle]*

For both recall periods, it was apparent that despite giving answers that were aligned to the recall period, the patients commonly explained that their answer(s) did not reflect their entire cycle.

***ID0014****: ‘Do you need to rest?’ I’d say yes. A little bit. During the past week I’d say quite a lot actually. Again, because of the cycle, this is a period, days 3 to 10, when I’m at my most tired, and I spent really the afternoon yesterday sleeping in the garden, just because I needed the rest, basically. [‘past week’ recall, ticked ‘quite a bit’] [male, 50–69 years old, urological cancer, first week of chemotherapy cycle]*

#### Construction of Averages

When responding to the questions with any recall period, most patients tended to attempt to provide averages over their entire cycle. This was because they typically experienced 1 week of low quality of life, 1 week of improved quality of life and 1 week of good quality of life (Fig. 2a, b). Averages were commonly constructed with the 4-week recall period, frequently occurred with the past week recall and occasionally occurred with ‘health today’. Some patients explicitly referred to providing averages to be able to reflect health-related quality of life over the past 4 weeks:

***ID0006****: It’s changing over three weeks, and you said four weeks, so that’s why I tried to respond on average. On average, it feels pretty good, but when the first week is there it feels pretty ghastly, and then you start to lose the will to live and forget a bit. Then you have the third cycle, but this third cycle does seem to be a bit tougher. [female, >70 years old, gynaecological cancer, second week of chemotherapy cycle]*

In other cases, many patients seemed to implicitly provide averages. Patients commonly described the cyclical nature of the symptoms when trying to decide which level of the attributes to choose. When attempting to average across the measures, there was a tendency for patients to report having problems ‘some of the time’ and for pain to interfere ‘a little bit’ on SF-12 (past 4 weeks) or ‘slight problems’ on EQ-5D (health today) and a ‘little bit’ affected on EORTC-QLQ-C30 (past week).

Implicit averaging was observed in the majority of the questions referring to the past 4 weeks.

***ID0026****: During the past four weeks, how much did your pain interfere with your normal work, including work outside the home and housework? I have four days, don't I, when it's really bad? Out of three weeks, I don't know how you would put four days into that. Would that be a little bit? I'm still alright, but it's just that I don't want to be doing much at all in those four days. No, and they have been all through the treatment, those four days. It's always been those four days. Then on the fourth day I'm alright again and then it just gets better and better through until the next treatment, doesn't it? I'll put 'a little bit', yes? [male, >70 years old, urological cancer, third week of chemotherapy cycle]*

For those few who did provide averages when thinking about their ‘health today’, ‘slight problems’ were commonly reported to provide ‘overall’ scores, as opposed to no problems or moderate/more severe problems.

***ID0007****: Pain and discomfort. ‘I have no pain or discomfort,’ slight pain, moderate pain. It depends, doesn’t it, which time of the month it can be almost? Overall, I’m not in great pain. ‘I have slight pain or discomfort,’ I would say. Yes. [female, 50–69 years old, gynaecological cancer, second week of chemotherapy cycle]*

***ID0020****: Self-care … Again, I can do most things for myself, get a bit tired … after a shower, or drying. So I would say again, probably ‘slight problems’ because, again, I don’t need any help yet, but on odd occasions I might do… [female, <50 years old, gynaecological cancer, third week of chemotherapy cycle]*

When thinking about the ‘past week’, many patients provided averages either over the week or over the treatment cycle due to the ups and downs.

***ID0030****: Have you felt nauseated? A little, during the beginning of the treatment. But, again, those tablets I take for nausea do make me tired, but I only took them for about three, four days following the chemo. [13 days post-chemotherapy when nausea has subsided] [male, 50–69 years old, colorectal cancer, second week of chemotherapy cycle]*

***ID0002****: Have you felt nauseated?’ Yes, a little, because I’m still getting sickness on and off. [female, >70 years old, gynaecological cancer, third week of chemotherapy cycle]*

#### Focus on the Worst Part or Underestimating Quality of Life

For both recall periods of ‘past week’ and ‘past 4 weeks’, some patients also focused on the worst part of their cycle for some questions despite saying in the semi-structured interview that they “usually feel fine except for one week where they feel terrible”. It was suggested that the worst parts were more salient than better parts of the cycle.

***ID0018****: “Yes, I think, yes, it’s not so easy going back four weeks, is it, really? That doesn’t really ... If you’re okay, it doesn’t stick in your mind so much, does it?” [female, >70 years old, colorectal cancer, second week of chemotherapy cycle]*

***ID0021****: In the last four weeks, how much has my pain interfered with my normal work? I automatically jump quite high with that [‘extremely’], especially because the first week I could barely get out of bed. My mind is driven by that thought rather than the slightly easier other two weeks. That’s a direct response to that first week rather than the second or third. [male, <50 years old, colorectal cancer, second week of chemotherapy cycle]*

***ID0030****: How much did pain interfere with normal work? Again, the early part of the treatment, it was very limiting, but after, say, a week, it was okay. So, I would say it was quite a bit to start with. [‘Quite a bit’ selected when completed at 13 days post-chemotherapy] [Male, 50–69 years old, colorectal cancer, second week of chemotherapy cycle]*

***ID0002****: [During the past week]‘Have you been constipated?’ Yes, a little bit. I always feel that, after the chemo, I do get constipated, yes. [21 days post-chemotherapy] [female, >70 years old, gynaecological cancer, third week of chemotherapy cycle]*

Most patients who focused on the worst part tended to be in the ‘better’ parts of the chemotherapy cycle, with only a few being in the worst part. Therefore, timing of assessment, whether in a good week or bad week, could influence whether patients referred to the worst part of their cycle (ESM 2).

#### Focus on the Best Part or Overestimating Quality of Life

Conversely, some patients ignored the worst parts of the cycle and focused on the best parts of the cycle. When completing the questions, the patients were either at the better parts of their cycle or had got through the worst part and were on an upward trajectory according to the pictorial task (ESM 3). These responses seemed to be partly due to adaptation to the health state because you “can’t always remember how bad you felt”, and because it was “too painful” to remember the difficult times as some patients did talk about not wanting to keep diaries of symptoms in the semi-structured interviews because they did not want to remember what it was like. The better parts of the cycle were referred to more commonly when thinking about the ‘past 4 weeks’.

***ID0008****: [four weeks] would have gone through a whole cycle of chemo as well. So it would have had the down bit and then feeling alright. I think probably I almost ignored the down bit, as opposed to … So on average over the four weeks it’s been fine. [female, 50–69 years old, gynaecological cancer, second week of chemotherapy cycle]*

***ID0027****: … You can always remember when you feel better, but sometimes you can’t always remember how bad you felt, three weeks after … this week it’s been alright, a little bit of a struggle, but, on the other weeks, sometimes you do forget how you are … [male, 50–69 years old, urological cancer, third week of chemotherapy cycle]*

#### Focus on Most Recent Feelings

Finally, patients were also more likely to refer to their most recent experiences when the recall period referred to the ‘past week’. This may have occurred because of the shorter recall period of the questionnaire and the specific referral to symptoms.

***ID0007****: ‘Have you felt nauseated?’ Yes, I was sick last night, which is very unusual, isn’t it? Yes, so I have a little. None of that, not like some people, I think. ‘Have you vomited?’ Yes, just a little … Only a little bit, only twice in the whole period have I been sick, yes. [female, 50–69 years old, gynaecological cancer, second week of chemotherapy cycle]*

***ID0006****: Okay, emotional problems. I did get very irritable over the last few days, I probably think that is emotional. I felt quite irritable, I got quite irritable with my Husband, so I’d say that was emotional, but it was just irritability, wasn’t depression or anything. So, some of the time. [female, >70 years old, gynaecological cancer, second week of chemotherapy cycle]*

#### Different Approaches Across and Within Questionnaires

When comparing the approaches to answering questions in a given questionnaire and across the different recall periods, it was clear that patients were using the different approaches, outlined previously, to answer the questions. For example, using quotes previously provided, when thinking about the ‘past week’ ID0002 provided averages for some questions and focused on the worst part of their cycle for other questions. ID0006 referred to the correct timeframe for some questions about ‘health today’, focused on most recent feelings for some questions about the ‘past week’ and constructed averages for some questions about the ‘past 4 weeks’. A few patients were aware that they were providing inconsistent answers, which could be because they were  trying to provide an overview of a fluctuating cycle, forgetting the recall period and finding certain aspects of the cycle to be more salient than others.

***ID0014****: [when completing the SF-12 with four week recall] I think I was thinking – I hope I was thinking – of more of the whole cycle, really, but I may have muddled that or not hit the absolute spot each time on that, possibly. [male, 50–69 years old, urological cancer, first week of chemotherapy cycle]*

***ID0006****: [reflecting on SF-12 and EORTC] Sometimes I was trying to respond on average, and I was aware sometimes it might have been inconsistent, but that doesn’t really matter does it, because I’m just responding as I feel, for those particular questions. [female, >70 years old, gynaecological cancer, second week of chemotherapy cycle]*

## Discussion

Many patients who experience fluctuations in health do not complete the recommended quality of life questionnaires according to the specified recall period. Both the recall period and the timing of assessment have an influence on patients’ responses. None of the recall periods were optimal for fluctuating health states. The shorter recall periods did not reflect all changes in quality of life and were likely to skew results causing an under or overestimation of quality of life depending on the timing of assessment. For both short and, more commonly, for longer recall periods, patients attempted to construct averages because of the number of changes that occurred within the recall time period or focused on specific points in the cycle, again influenced by timing of assessment. Patients were not systematic in the way they answered the questions, as they employed different approaches when answering different questions across questionnaires and even within the same recall period. Consequently, the current use or meaningfulness of these types of responses and the subsequent calculation of health state values in these conditions must be questioned when used in economic evaluations to inform resource allocation decisions. Although studied here in relation to cancer chemotherapy, these findings are likely to have general applicability to other areas of recurrent fluctuating health states, particularly where these fluctuations are, as in cancer chemotherapy, relatively predictable.

### Strengths and Limitations

To our knowledge, this is the first study to determine how patients with recurrent fluctuating symptoms complete questionnaires with different recall periods and how patients’ approaches to completing recommended measures can be influenced by the timing of the assessment. The sample included a broad range of chemotherapy regimes and a range of commonly used questionnaire recall periods. Whilst the heterogeneity between patients with respect to treatment type could be thought to limit the generalisability of the findings, saturation was reached in the themes expressed by the patients and it is therefore unlikely that a different sample would produce different findings. However, it is acknowledged that think-aloud interviews rely on participants verbalising their thoughts and this process could have made participants think more carefully about the recall period and the questions. Finally, although all participants were recruited from the same oncology centre, it is a tertiary regional centre that follows national guidance on the management of cancer and therefore the cyclical nature of the treatments provided do not differ by setting. To mitigate against any potential bias, the study did not include patients who were receiving treatments on clinical trials.

### Comparison with Other Studies

To our knowledge, no other study has used qualitative interviews to understand how patients complete questionnaires when health fluctuates. Other studies have discussed completion of questionnaires more generally, for example, one quantitative study compared a 1-week recall period to ‘health today’ in resolved serious adverse events and concluded that health utility in the 1-week recall period was lower than for health today. The authors interpret this finding to mean that the recall period was adhered to and discuss that fluctuations in health may occur in recall periods longer than a day and if this occurred patients may construct an average, focus on recovery or poor health [31]. Assuming respondents adhere to the recall period, the short recall period of EQ-5D (‘health today’) could result in changes in quality of life being missed [31]. Thus, if the respondent experiences a change in health state during the recall period, it is not clear what is reported [31]. Studies in the psychology and behavioural economics literature have also quantitatively assessed completion and suggest that patients’ memories, and their response to questionnaires with retrospective recall periods, are determined by the worst and end part of an episode (‘peak-end rule’) as patients’ judgement of pain was strongly correlated with the peak pain intensity and pain at the end of a procedure [32], though anticipatory emotions and the speed of changes can also play a part [33]. The current study shows that when completing these questionnaires there is a tendency to observe aspects of the peak-end rule when using SF-12 and EORTC-QLQ-C30, but that patients are also constructing averages and thinking about the best part of their cycle.

### Implications for Policy

Our study shows that the recall period, timing of assessment and the assumptions about quality of life estimations that underlie the QALY calculation may not be appropriate for recurrent fluctuating health states. Therefore, an intervention that may not offer the best value for money could be inadvertently recommended to decision makers for practice and implemented. The combination of recurrent fluctuations in health and current methods of assessing quality of life seem to lead to inconsistent answers when completing quality of life questions within the same questionnaire and across questionnaires. Responses to currently recommended questionnaires thus may not necessarily reflect a patient’s quality of life trajectory when health is fluctuating.

When assessing quality of life using current approaches to inform resource allocation and make treatment decisions, quality of life and QALYs may be over or underestimated if the recommended measure, EQ-5D, is used because of the cyclical fluctuations. Therefore, drugs could be shown to offer greater value for money due to the measurement of quality of life and calculation of QALYs than is accurate, and vice versa. This is particularly the case for trials of chemotherapy, where quality of life measures are typically administered on the first day of each chemotherapy cycle when patients are feeling their best [34]. In this case, any differences in side-effect profiles (and therefore quality of life) would not be detected by the measures and the analysis biased against drugs with better side-effect profiles. When using the next recommended alternative in the UK, SF-12, or even the commonly used cancer-specific measure, EORTC-QLQ-C30, patients are using different approaches to complete the questionnaire. For any of these measures, the answers provided by patients may not reflect what the questionnaire intends to capture and it is not clear how meaningful it is to use such questionnaires in their current form when health fluctuates to capture quality of life and calculate QALYs when responses are inconsistent; one implication for practice may be that greater patient involvement early in the study design of trials and economic evaluations could help to identify some of these issues. This finding is likely to be a broader concern than in the area of chemotherapy alone, as there are many recurrent fluctuating conditions or treatments, such as epilepsy, bipolar disorder and multiple sclerosis. The direction of any potential bias is unclear from this research and further research is required to determine the extent to which these approaches cause misleading results. ISPOR guidance [35] suggests a general awareness of difficulties in capturing quality of life and the importance of collecting data that reflect the health state experienced, but it remains very unclear what the optimum strategy is for obtaining these data. Whilst setting recall periods to reflect the length of the chemotherapy cycle (here) or the average period within which fluctuations are experienced (in other settings), might seem the obvious strategy, the data here suggest some individuals find this averaging challenging and it is an empirical question as to whether the approach of asking people to do this averaging themselves, or adapting an alternative approach, is likely to be preferable. For fluctuating conditions, where quality of life is constantly changing, this might involve eliciting information from patients about different periods of time (or aspects of their entire experience) during a fluctuation, and the duration of these states that can then be applied across time. Further research should explore ‘how’ and ‘when’ to ask questions about quality of life when health fluctuates recurrently by changing recall periods and assessing the influence of different assessment timings on results.

## Conclusions

By gaining a better understanding of how patients with fluctuating health complete questionnaires with different recall periods at different points in time, this research goes beyond anecdotal reports and presents evidence that patients do not complete questionnaires systematically or complete them as researchers expect because of the fluctuations they experience in health. The evidence on their reasoning presented in this article can be used to adapt current methods or develop more appropriate methods and techniques to capture quality of life and calculate QALYs.

## Supplementary Information

Below is the link to the electronic supplementary material.

Supplementary file1 (PDF 305 kb)

Supplementary file2 (PDF 184 kb)

Supplementary file3 (PDF 211 kb)

## Declarations

### Availability of data and material

The datasets generated during and/or analysed during the study are not available to protect the anonymity of study participants.
