# Supplementary material for: Challenges in Using Recommended Quality of Life Measures to Assess Fluctuating Health: A Think-Aloud Study to Understand How Recall and Timing of Assessment Influence Patient Responses
Source: Patient. 2021 Dec 2;15(4):445–57. doi: 10.1007/s40271-021-00555-7 (PMC9197908; doi:10.1007/s40271-021-00555-7)
Supplement: Supplementary file 1 — Supplementary file1 (PDF 305 kb) [file 40271_2021_555_MOESM1_ESM.pdf]

Online Resource 1

Title: Challenges in using recommended quality of life measures to assess fluctuating health: a think-aloud study to understand how recall and timing of assessment influence patient responses

Short running title: Completion of measures when health fluctuates

Journal: The Patient

Authors:

Sabina Sanghera[1*], Axel Walther[2], Tim J Peters[3], Joanna Coast[1]

[1]Health Economics Bristol, Population Health Sciences, Bristol Medical School, University of Bristol, Bristol, BS8 1NU, UK

[2]Bristol Cancer Institute, University Hospitals Bristol NHS Foundation Trust

[3]Population Health Sciences, Bristol Medical School, University of Bristol

***Corresponding Author:**

Dr Sabina Sanghera

Health Economics Bristol (HEB),

Population Health Sciences, Bristol Medical School

University of Bristol

1-5 Whiteladies Road

Bristol

BS8 1NU

Telephone: 0117 428 3124

Email: sabina.sanghera@bristol.ac.uk

Extract of early analytic account

Analytic account (6,10,18)

INTERVIEWS 6,10, 18

The interviews in this descriptive comprised three female patients aged between 70-85 years old who were undergoing 3 weekly chemotherapy cycle regimens.

ID 6: cycle number 3 and 8 days since chemotherapy

ID 10: cycle number 5 and 7 days since chemotherapy

ID 18: cycle number 3 and 8 days since chemotherapy

This account consists of two types of interview: (1) a think-aloud interview that was carried out alongside the completion of three questionnaires, EQ-5D, SF-12 and EORTC, and (2) a semi-structured interview, which followed the think-aloud interview and included a pictorial task where patients were asked to draw how their quality of life changed during one cycle of chemotherapy. It should be noted that in the think-aloud aspect of the interview patients are providing responses to items on a questionnaire and therefore the answers are prompted.

1) Cyclical nature of side effects:

The patients described the chemotherapy as being cyclical. Patients were consistent in describing their quality of life as changing throughout the cycle: the first few days after chemotherapy were described as having relatively high quality off, followed by a decline in health and then patients thought their quality of life began to improve back towards 'normal' in the final week before beginning the next cycle of chemotherapy.

> *ID0010: I am fine when I start the chemo. Then it is fine for a few days. Then it starts going down. It goes down and it goes on then for a few weeks. Then I start picking up again. When I am ready for the next lot I am okay again.*

> *ID0018: So, I have the chemo and it's okay for a few days, then I'm not too good, and then I gradually improve (…) by next week [the final week before treatment], I will feel a lot better …, which is what happens the last couple of times that I felt quite well, in fact. You could almost forget you had cancer.*

The patients also drew their changes in quality of life in a pictorial task and in all three cases the patients suggest that quality of life declines and returns to a high level at the end of the cycle. It is at the end/beginning of the cycle when quality of life questionnaires are typically administered.

ID0010

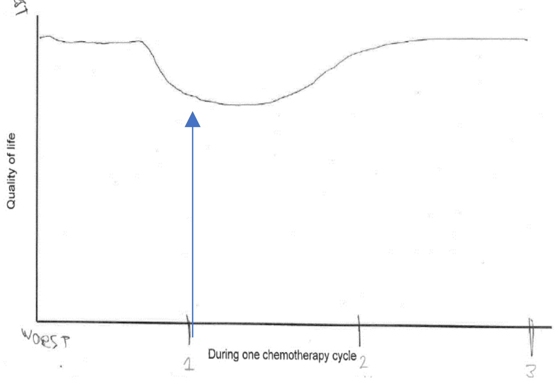

ID0018

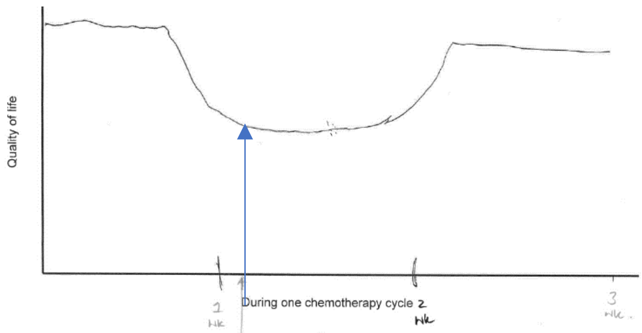

ID006

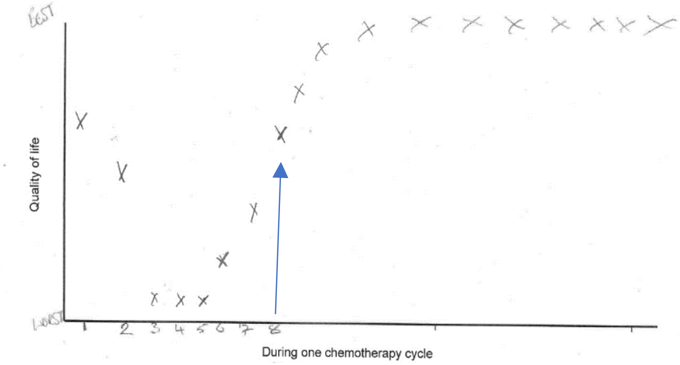

In slight contrast to the other patients, one patient described the cyclical nature, but noticed a much faster decline in quality of life.

> *ID 0006 So, we have the day after, which is one which, I seem to be able to do things on the day after (…), I mean not as I would normally be, but it's not too bad so maybe that's about there. Then day two doesn't seem to be too bad. It's probably about there, and then every week seems to drop…So, it goes quite low there, so that's the Thursday, Friday, Saturday, Sunday, it's probably quite low on the Monday. It starts to get a bit better there. Thursday, Friday, Saturday, Sunday, Monday, Tuesday, Wednesday. This is Thursday. Then I think it just stays up from the previous experience of the two cycles.*

1.1) COMPLETION OF THE QUESTIONNAIRES:

Each patient experienced a change in quality of life in the past 7 days with health declining in the most recent three days when completing the questionnaires. It appeared that difficulties arose when patients were completing the questionnaires due to the cyclical nature of the symptoms. There seemed to be inconsistencies in responses when answering questions on SF-12 and EORTC due to the recall periods of past 4 weeks and past week, respectively. Some patients provided average values and in other cases appeared to adopt the peak-end rule.

1.11. Think-aloud with the SF-12 (Past 4 weeks)

One patient changed the timeframe and another described difficulty in completing the questionnaire. Both of their reasons seemed to be due to the mismatch in the three-weekly cycle of the chemotherapy and the four weeks recall period of the questionnaire.

> *ID0010: That is difficult. I'm not really sure about that to be honest. Over the past four weeks, with that it sort of goes up and down…. Before that, it would have been about a week before I went to the hospital that I would have felt a bit low. It [the cycle] is every three weeks, so you can't really say four weeks.*

> *ID0006: The past four weeks? …If you call it three weeks, and I'm saying that the second weeks not too bad, the third weeks almost normal, that's why I'm calling it some of the time.*

Average

Having described their symptoms as being cyclical in both the semi-structured interviews and pictorial task, when completing SF-12, patients seemed to report problems 'some of the time' for dimensions that refer to the past 4 weeks. In one case the patient explicitly explained that she used averages and explained that she tried to provide average values to reflect her health-related quality of life over the past four weeks:

> *ID 0006 It's changing over three weeks, and you said four weeks, so that's why I tried to respond on average. On average, it feels pretty good, but when the first week is there it feels pretty ghastly, and then you start to lose the will to live and forget a bit. Then you have the third cycle, but this third cycle does seem to be a bit tougher. A little bit tougher I would say.*

In other cases, patients seemed to implicitly use averages. This was identified by comparing the pictorial task, what was said during the semi-structured interview and what was reported in the questionnaire. After explaining that she can 'get on with things, normally' in the semi-structured interview, the patient ticked that she was having difficulties with regular activities 'some of the time' in the questionnaire.

> *ID0010: Yes, definitely. I have got more energy than this normally. I can get on and do sewing and things and go out. I just haven't felt like it. It was a […] meeting last night and I didn't go. I thought, "I can't go and sit through that."*

> *ID0010:* During the past four weeks have you had any of the following problems with your work or other regular activities as a result of your physical health? Some of the time, I would say. Were you limited in the kind of work or other activities? Yes, some of the time.

Similarly, after explaining in the pictorial task that in the last week of the cycle “you could almost forget you had cancer”, when answering questions about daily activities, the patient seemed to provide averages

> *ID0018:* ‘Have you had any of the following problems with your work or other regular daily activities?’ Yes, definitely, I’ve accomplished less than I would like some of the time. ‘Were limited in, kind of, work or other activities?’ Yes, some of the time

Peak end rule - most recent feelings

When the patient who explicitly provided average values was asked to provide a rating for her global health (health overall), she considered quality of life over three weeks, but seemed to provide an answer for her most recent feelings

> *ID 0006: It seems to, affect me differently in the different parts of the cycle. So, I’d sort of say, the first week is ghastly, but it’s not that ghastly. The second weeks’ ok, and the third week becomes very good. So, let’s just tick very good.*

Again, when completing over the past four weeks she appeared to answer in response to the last few days

> *ID 0006: Okay, emotional problems. I did get very irritable over the last few days, I probably think that is emotional. I felt quite irritable, I got quite irritable with my Husband, so I’d say that was emotional, but it was just irritability, wasn’t depression or anything. So, some of the time.*

Peak end rule - worst part

When completing the questionnaire the patient ticked the worst level on the questionnaire, but later in the semi-structured interview they said they usually feel fine except for one week they feel terrible and suggested that they were focusing on the worst part because these moments are more memorable than better parts of the cycle.

- *ID0018: “Yes, I think, yes, it’s not so easy going back four weeks, is it, really? That doesn’t really- ... If you’re okay, it doesn’t stick in your mind so much, does it?”*
