# Supplementary material for: Challenges in Using Recommended Quality of Life Measures to Assess Fluctuating Health: A Think-Aloud Study to Understand How Recall and Timing of Assessment Influence Patient Responses
Source: Patient. 2021 Dec 2;15(4):445–57. doi: 10.1007/s40271-021-00555-7 (PMC9197908; doi:10.1007/s40271-021-00555-7)
Supplement: Supplementary file 2 — Supplementary file2 (PDF 184 kb) [file 40271_2021_555_MOESM2_ESM.pdf]

Online Resource 2

Title: Challenges in using recommended quality of life measures to assess fluctuating health: a think-aloud study to understand how recall and timing of assessment influence patient responses

Short running title: Completion of measures when health fluctuates

Journal: The Patient

Authors:
Sabina Sanghera[1*], Axel Walther[2], Tim J Peters[3], Joanna Coast[1]

[1]Health Economics Bristol, Population Health Sciences, Bristol Medical School, University of Bristol, Bristol, BS8 1NU, UK

[2]Bristol Cancer Institute, University Hospitals Bristol NHS Foundation Trust

[3]Population Health Sciences, Bristol Medical School, University of Bristol

***Corresponding Author:**
Dr Sabina Sanghera
Health Economics Bristol (HEB),
Population Health Sciences, Bristol Medical School
University of Bristol
1-5 Whiteladies Road
Bristol
BS8 1NU
Telephone: 0117 428 3124
Email: sabina.sanghera@bristol.ac.uk

Figure S1. Patients who focused on the worst part of their cycle

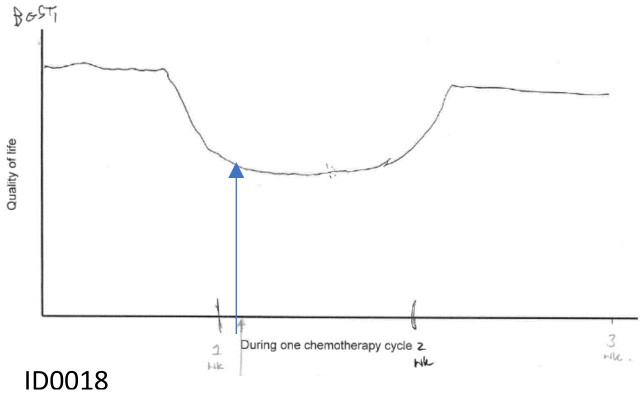

ID0018

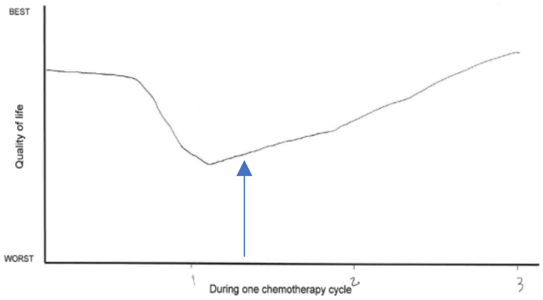

ID0028

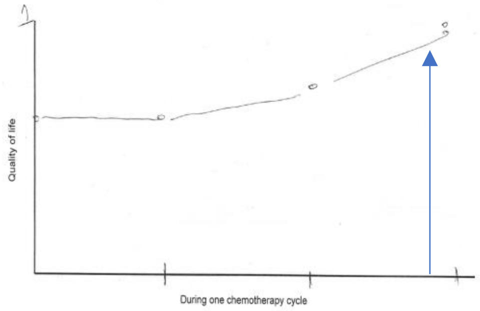

ID0011

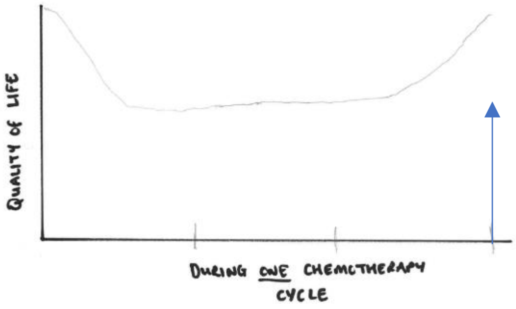

ID0002

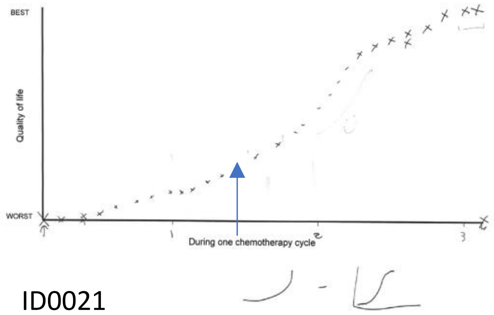

ID0021

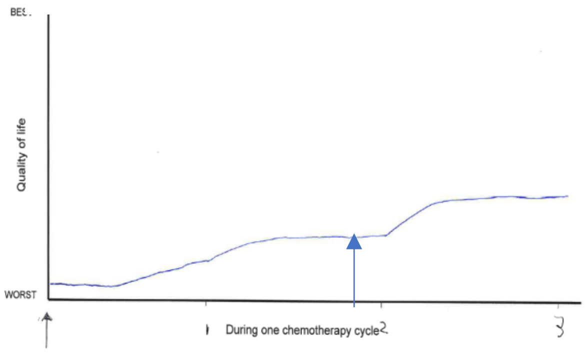

ID ID0030 x higher up on the graph.

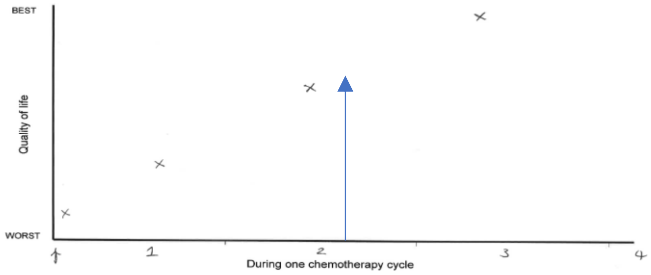

ID0020
