# Supplementary material for: Challenges in Using Recommended Quality of Life Measures to Assess Fluctuating Health: A Think-Aloud Study to Understand How Recall and Timing of Assessment Influence Patient Responses
Source: Patient. 2021 Dec 2;15(4):445–57. doi: 10.1007/s40271-021-00555-7 (PMC9197908; doi:10.1007/s40271-021-00555-7)
Supplement: Supplementary file 3 — Supplementary file3 (PDF 211 kb) [file 40271_2021_555_MOESM3_ESM.pdf]

Online Resource 3

Title: Challenges in using recommended quality of life measures to assess fluctuating health: a think-aloud study to understand how recall and timing of assessment influence patient responses

Short running title: Completion of measures when health fluctuates

Journal: The Patient

Authors:

Sabina Sanghera[1*], Axel Walther[2], Tim J Peters[3], Joanna Coast[1]

[1]Health Economics Bristol, Population Health Sciences, Bristol Medical School, University of Bristol, Bristol, BS8 1NU, UK

[2]Bristol Cancer Institute, University Hospitals Bristol NHS Foundation Trust

[3]Population Health Sciences, Bristol Medical School, University of Bristol

***Corresponding Author:**

Dr Sabina Sanghera

Health Economics Bristol (HEB),

Population Health Sciences, Bristol Medical School

University of Bristol

1-5 Whiteladies Road

Bristol

BS8 1NU

Telephone: 0117 428 3124

Email: sabina.sanghera@bristol.ac.uk

Figure S2. Patients who focused on the best part of their cycle.

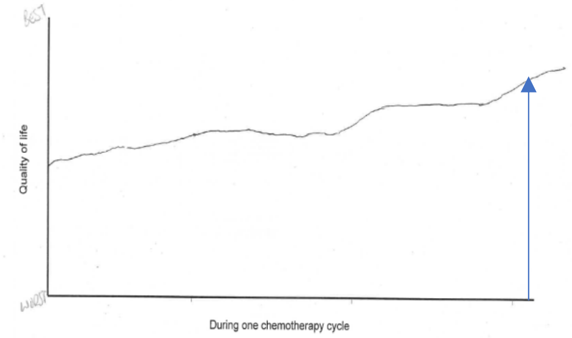

ID0003

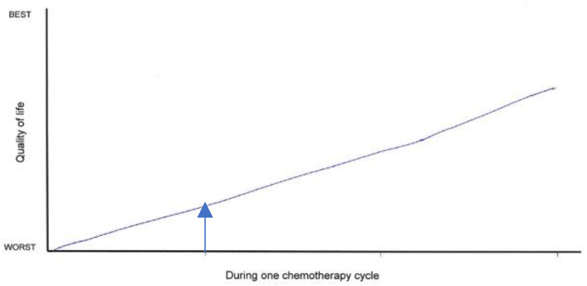

ID0009

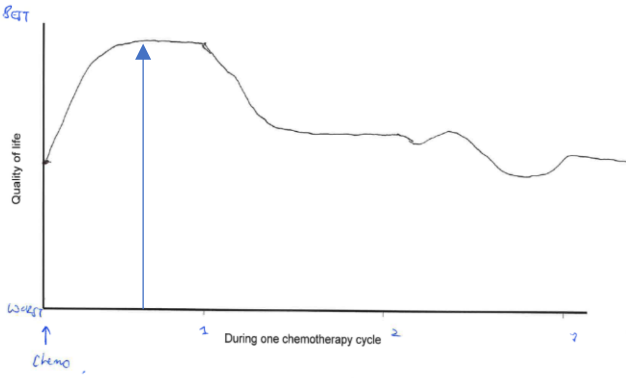

ID0007

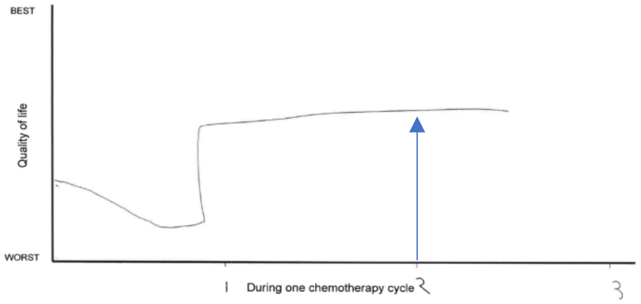

ID0008

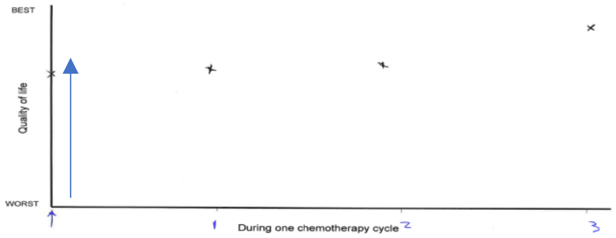

ID0022

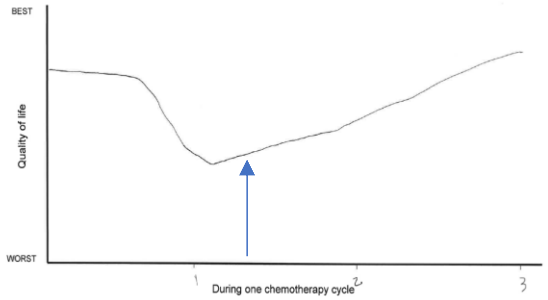

ID0028

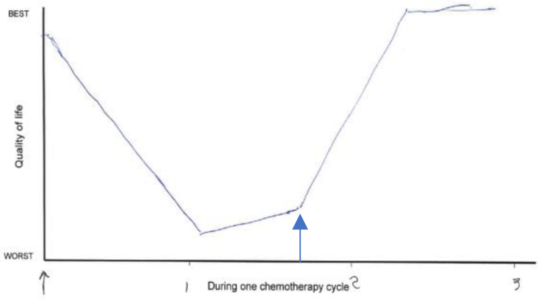

ID0015
